# Analysis of *Poncirus polyandra* (Rutaceae) chloroplast genome and its phylogenetic implications

**DOI:** 10.1080/23802359.2019.1627928

**Published:** 2019-07-11

**Authors:** Yang Yang, Hong-Zhi Wu, Li-Ming Zhao, Shui-Lian He

**Affiliations:** a Yunnan Key Laboratory of Biomass Big Data, Yunnan Agricultural University, Kunming, China;; b College of Science, Yunnan Agricultural University, Kunming, China;; c College of Horticulture and Landscape, Yunnan Agricultural University, Kunming, China;; d Yunnan Academy of Forestry, Kunming, China

**Keywords:** *Poncirus polyandra*, endangered species, chloroplast genome, phylogenetic analysis

## Abstract

*Poncirus polyandra* is a threatened plant in China Now, the complete chloroplast (cp) genome of *P. polyandra* was assembled. The cp genome of *P. polyandra* was 160,212 bp in length, it consists of a pair of inverted repeats ((IRa and IRb) regions (27,016 bp) separated by the large single-copy (LSC, 87,407 bp) and small single-copy (SSC, 18,775 bp) regions. The cp genome encodes 105 unique genes, including 70 protein-coding genes, 30 transfer RNA genes, 4 ribosomal RNA genes, and 1 pseudogene. The phylogenetic tree of Rutaceae showed that *P. polyandra* was clustered together with genus *Citrus* and *Poncirus.*

*Poncirus polyandra* S. Q. Ding et al. is a small evergreen tree, which belongs to the genus *Poncirus* of the family Rutaceae. The species is known to be sparsely distributed only in limestone habitats of Fuming counties in Yunnan Province of China. In recent years, the natural habitat of *P. polyandra* is severely damaged by over-exploitation. It is an extremely small population species endemic to Yunnan province. In the present study, applying the Illumina technology, the whole chloroplast genome of *P. polyandra* was sequenced, assembled, and annotated. Understanding the chloroplast genome information of this rare and endangered tree may provide valuable guidelines for the development of management strategies for both *in situ* and *ex situ* conservation activities (Duan et al. 2017).

The fresh leaves of *P. polyandra* were collected from the arboretum of Chinese Academy of Forestry (25.16° N, 102.75° E). The voucher specimen was deposited at Herbarium, Kunming Institute of Botany, CAS (KUN). Total genomic DNA was isolated from fresh leaves using a DNeasy Plant Mini Kit (QIAGEN, Valencia, California, USA) according to the manufacturer’s instructions to construction chloroplast DNA libraries. The Illumina sequencing was conducted by Shanghai Genesky Biotechnologies Inc. (Shanghai, China). Resultant clean reads were assembled using GetOrganelle pipeline (https://github.com/Kinggerm/GetOrganelle). The genome was automatically annotated by using the CpGAVAS pipeline (Liu et al. 2012) and start/stop codons and intron/exon boundaries were adjusted in Geneious R11.0.2 (Biomatters Ltd., Auckland, New Zealand). All the contigs were checked against the reference genome of *Citrus maxima* (NC034290).

The complete cp genome sequence was submitted to the GenBank to get the accession number MK764539 and was 160,212 bp in length. It was the typical quadripartite structure and contained two short inverted repeat (IRa and IRb) regions (27,016 bp) which were separated by a small single copy (SSC) region (18,775 bp) and a large single copy (LSC) region (87,407 bp). The cp genome encodes 105 unique genes, including 70 protein-coding genes, 30 transfer RNA (tRNA) genes, 4 ribosomal RNA (rRNA) genes, and 1 pseudogene. Twenty-one gene species are partially or completely duplicated, including nine PCG (*ndhB*, *ndhF*, *rpl2*, *rpsl23*, *rps7*, *rps12*, *rps19*, *ycf1*, and *ycf2*), seven tRNA (*trnI-GAU*, *trnA-UGC*, *trnL-CAA*, *trnI-CAU*, *trnR-ACG*, *trnV-GAC*, and *trnN-GUU*), all four rRNA (4.5S, 5S, 16S, and 23S rRNA), and one pseudogene (*Ψrpl22*). The AT content of the cp genome was 61.6%, while the corresponding values for LSC, SSC, and IR regions were 63.2, 64.8, and 57.1%, respectively.

The chloroplast genome sequences of Rutaceae were downloaded from GenBank and aligned with *P. polyandra* using MAFFT (Katoh and Standley 2013) in Geneious R11.0.2 (Auckland, New Zealand). To resolve its phylogenetic placement within the family Rutaceae, the maximum-likelihood (ML) phylogeny tree was reconstructed using RAxML version 8.1.1179 (Stamatakis 2014). *Commiphora wightii* (NC036978, Burseraceae) was selected as an outgroup. The topology of the phylogenetic tree showed that the species of *P. polyandra* was clustered together with genus *Citrus* and *Poncirus* (Figure 1). The complete cp genome information reported in this study will be a valuable resource for future studies of the species’ genetic diversity, and the conservation of this highly endangered species.

**Figure 1.**
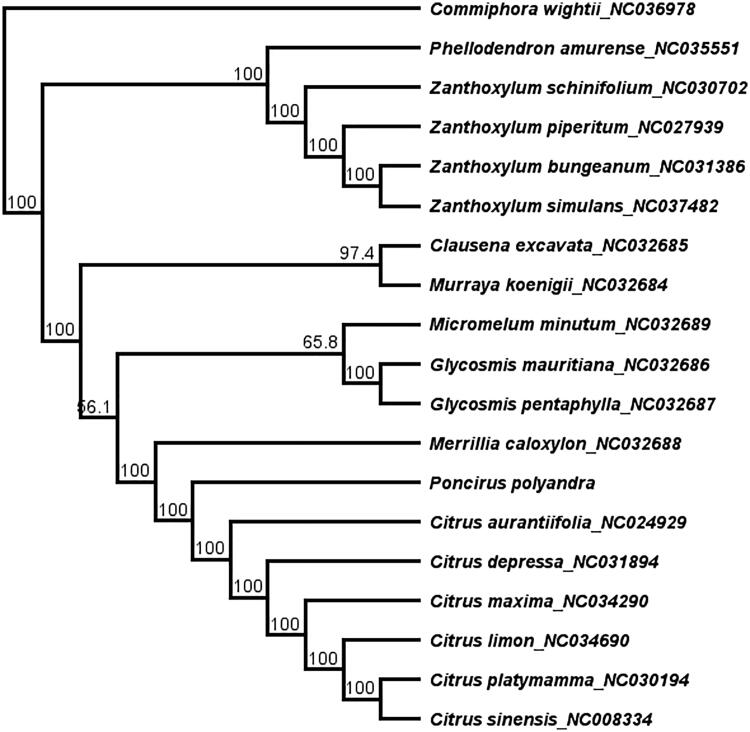
The maximum-likelihood (ML) phylogenetic tree based on 19 complete chloroplast genome sequence. Numbers at the right of nodes are bootstrap support values.
